# Supplementary material for: Hot spot prediction in protein-protein interactions by an ensemble system
Source: BMC Syst Biol. 2018 Dec 31;12(Suppl 9):132. doi: 10.1186/s12918-018-0665-8 (PMC6311905; doi:10.1186/s12918-018-0665-8)
Supplement: Supplementary file 2 — All relevant MATLAB code. A simple Matlab implement of our predictor. (ZIP 19,742 kb) [file 12918_2018_665_MOESM2_ESM.zip › Additional file 2/Read ASA/ASA Property/SKEMPI/2B42A.txt]

 NetSurfP - Protein Surface Accessibility and Secondary Structure Predictions

  

|  |  |  |
| --- | --- | --- |
|  |  | NetSurfP - Protein Surface Accessibility and Secondary Structure Predictions  Technical University of Denmark |

  


---

```
# For publication of results, please cite:
# A generic method for assignment of reliability scores applied to solvent accessibility predictions.
# Bent Petersen, Thomas Nordahl Petersen, Pernille Andersen, Morten Nielsen and Claus Lundegaard
# BMC Structural Biology 2009, 9:51 doi:10.1186/1472-6807-9-51
#
# Column 1: Class assignment - B for buried or E for Exposed - Threshold: 25% exposure, but not based on RSA
# Column 2: Amino acid
# Column 3: Sequence name
# Column 4: Amino acid number
# Column 5: Relative Surface Accessibility - RSA
# Column 6: Absolute Surface Accessibility
# Column 7: Z-fit score for RSA prediction
# Column 8: Probability for Alpha-Helix
# Column 9: Probability for Beta-strand
# Column 10: Probability for Coil
E L  2B42A                  1    0.480  87.851  -0.504   0.003   0.003   0.994
E P  2B42A                  2    0.315  44.670  -1.024   0.004   0.085   0.910
B V  2B42A                  3    0.168  25.822  -0.619   0.004   0.616   0.381
B L  2B42A                  4    0.201  36.876  -0.366   0.002   0.816   0.182
B A  2B42A                  5    0.139  15.340   0.037   0.002   0.816   0.182
B P  2B42A                  6    0.243  34.425  -0.501   0.021   0.756   0.223
B V  2B42A                  7    0.069  10.605   0.069   0.004   0.616   0.381
E T  2B42A                  8    0.308  42.720  -0.282   0.004   0.514   0.481
E K  2B42A                  9    0.414  85.242  -0.572   0.005   0.262   0.733
E D  2B42A                 10    0.337  48.619  -0.923   0.004   0.138   0.858
E P  2B42A                 11    0.446  63.358  -2.047   0.018   0.047   0.935
E A  2B42A                 12    0.445  49.083  -2.017   0.005   0.045   0.951
E T  2B42A                 13    0.308  42.692  -0.450   0.004   0.138   0.858
B S  2B42A                 14    0.081   9.528  -0.856   0.004   0.616   0.381
B L  2B42A                 15    0.107  19.518  -0.120   0.001   0.900   0.099
B Y  2B42A                 16    0.064  13.762   0.304   0.001   0.959   0.040
B T  2B42A                 17    0.116  16.061   0.343   0.001   0.959   0.040
B I  2B42A                 18    0.031   5.735   0.053   0.001   0.959   0.040
B P  2B42A                 19    0.257  36.482   0.719   0.001   0.959   0.040
B F  2B42A                 20    0.049   9.754   0.181   0.001   0.900   0.099
E H  2B42A                 21    0.301  54.843  -0.395   0.003   0.718   0.279
B D  2B42A                 22    0.179  25.852  -1.395   0.004   0.514   0.481
E G  2B42A                 23    0.276  21.721  -0.983   0.019   0.141   0.840
B A  2B42A                 24    0.239  26.283  -0.337   0.018   0.047   0.935
B S  2B42A                 25    0.228  26.745  -0.145   0.022   0.359   0.619
B L  2B42A                 26    0.045   8.185   0.722   0.018   0.846   0.136
B V  2B42A                 27    0.085  13.003   0.251   0.011   0.918   0.071
B L  2B42A                 28    0.038   7.049  -0.248   0.011   0.918   0.071
B D  2B42A                 29    0.042   6.052  -0.879   0.023   0.655   0.322
B V  2B42A                 30    0.051   7.885  -0.104   0.004   0.197   0.799
B A  2B42A                 31    0.111  12.177  -0.935   0.005   0.045   0.951
B G  2B42A                 32    0.080   6.296  -0.594   0.018   0.047   0.935
B P  2B42A                 33    0.192  27.287  -0.986   0.018   0.088   0.893
B L  2B42A                 34    0.154  28.142  -0.633   0.022   0.359   0.619
B V  2B42A                 35    0.030   4.642   0.179   0.023   0.655   0.322
B W  2B42A                 36    0.032   7.816   0.176   0.018   0.846   0.136
B S  2B42A                 37    0.029   3.422   0.240   0.018   0.846   0.136
B T  2B42A                 38    0.116  16.117   0.075   0.021   0.756   0.223
B C  2B42A                 39    0.048   6.711  -1.264   0.004   0.197   0.799
E K  2B42A                 40    0.479  98.530  -0.413   0.005   0.045   0.951
E G  2B42A                 41    0.624  49.101  -0.561   0.005   0.015   0.979
B E  2B42A                 42    0.121  21.139  -1.167   0.005   0.045   0.951
E I  2B42A                 43    0.504  93.259  -1.047   0.018   0.047   0.935
E P  2B42A                 44    0.560  79.450  -0.394   0.018   0.047   0.935
B C  2B42A                 45    0.140  19.600  -1.298   0.018   0.019   0.964
E S  2B42A                 46    0.547  64.108  -1.556   0.018   0.047   0.935
E S  2B42A                 47    0.493  57.744  -1.047   0.018   0.047   0.935
E P  2B42A                 48    0.500  70.907  -0.920   0.018   0.047   0.935
E T  2B42A                 49    0.444  61.597  -1.241   0.018   0.019   0.964
B C  2B42A                 50    0.151  21.243  -1.733   0.018   0.047   0.935
E L  2B42A                 51    0.542  99.259  -1.063   0.018   0.088   0.893
E L  2B42A                 52    0.463  84.757  -1.466   0.019   0.141   0.840
E A  2B42A                 53    0.326  35.936  -1.173   0.019   0.141   0.840
E N  2B42A                 54    0.541  79.232  -2.097   0.019   0.141   0.840
E A  2B42A                 55    0.420  46.273  -1.005   0.004   0.138   0.858
B Y  2B42A                 56    0.224  47.954  -1.919   0.004   0.138   0.858
E P  2B42A                 57    0.542  76.924  -0.715   0.005   0.045   0.951
E A  2B42A                 58    0.407  44.818  -1.608   0.005   0.015   0.979
E P  2B42A                 59    0.590  83.749  -2.065   0.018   0.019   0.964
E G  2B42A                 60    0.545  42.884  -1.135   0.018   0.019   0.964
B C  2B42A                 61    0.164  23.068  -3.583   0.018   0.019   0.964
E P  2B42A                 62    0.509  72.156  -1.564   0.018   0.088   0.893
E A  2B42A                 63    0.524  57.789  -0.793   0.019   0.141   0.840
E K  2B42A                 64    0.383  78.721  -0.730   0.019   0.141   0.840
E P  2B42A                 65    0.608  86.233  -0.476   0.018   0.088   0.893
E C  2B42A                 66    0.463  65.061  -1.787   0.004   0.085   0.910
E T  2B42A                 67    0.414  57.450  -0.443   0.004   0.085   0.910
E A  2B42A                 68    0.484  53.359  -1.136   0.005   0.262   0.733
B Y  2B42A                 69    0.133  28.379   0.699   0.004   0.514   0.481
E P  2B42A                 70    0.443  62.918  -0.372   0.004   0.616   0.381
B Y  2B42A                 71    0.112  23.934   0.006   0.002   0.816   0.182
E N  2B42A                 72    0.364  53.216  -0.627   0.002   0.816   0.182
B P  2B42A                 73    0.159  22.491  -1.127   0.022   0.552   0.426
B V  2B42A                 74    0.292  44.850  -2.288   0.020   0.205   0.775
E S  2B42A                 75    0.434  50.877  -1.495   0.004   0.138   0.858
B G  2B42A                 76    0.267  21.044  -2.261   0.004   0.138   0.858
E A  2B42A                 77    0.393  43.342  -1.533   0.005   0.262   0.733
B C  2B42A                 78    0.205  28.852  -1.192   0.004   0.616   0.381
B A  2B42A                 79    0.103  11.307  -1.125   0.004   0.616   0.381
B A  2B42A                 80    0.217  23.880  -1.059   0.003   0.718   0.279
B G  2B42A                 81    0.167  13.135  -0.117   0.002   0.816   0.182
B S  2B42A                 82    0.172  20.182  -0.954   0.002   0.816   0.182
B L  2B42A                 83    0.157  28.820  -0.214   0.002   0.816   0.182
B S  2B42A                 84    0.083   9.692  -1.838   0.001   0.900   0.099
E H  2B42A                 85    0.267  48.622   0.141   0.002   0.816   0.182
B T  2B42A                 86    0.074  10.292  -0.821   0.003   0.718   0.279
B R  2B42A                 87    0.196  44.838   0.555   0.003   0.718   0.279
B F  2B42A                 88    0.044   8.871   0.347   0.001   0.900   0.099
B V  2B42A                 89    0.121  18.644  -0.640   0.001   0.959   0.040
B A  2B42A                 90    0.045   4.970  -0.050   0.001   0.900   0.099
B N  2B42A                 91    0.185  27.025  -1.124   0.021   0.451   0.528
B T  2B42A                 92    0.317  43.954  -1.660   0.021   0.279   0.699
B T  2B42A                 93    0.300  41.679  -1.365   0.004   0.420   0.576
E D  2B42A                 94    0.486  70.105  -1.080   0.022   0.359   0.619
B G  2B42A                 95    0.188  14.788  -1.425   0.021   0.279   0.699
E S  2B42A                 96    0.489  57.276  -0.912   0.019   0.141   0.840
E K  2B42A                 97    0.392  80.717  -1.113   0.056   0.142   0.802
E P  2B42A                 98    0.335  47.565  -1.249   0.064   0.216   0.721
E V  2B42A                 99    0.308  47.340  -1.393   0.021   0.451   0.528
B S  2B42A                100    0.269  31.492  -1.023   0.022   0.552   0.426
E K  2B42A                101    0.306  62.924  -1.136   0.022   0.552   0.426
B V  2B42A                102    0.199  30.602  -0.638   0.023   0.655   0.322
B N  2B42A                103    0.244  35.648  -0.636   0.022   0.552   0.426
B V  2B42A                104    0.297  45.603  -0.459   0.074   0.484   0.442
E G  2B42A                105    0.419  32.991  -0.933   0.069   0.386   0.545
B V  2B42A                106    0.176  27.097  -0.763   0.022   0.552   0.426
B L  2B42A                107    0.204  37.426  -0.591   0.021   0.756   0.223
B A  2B42A                108    0.148  16.277  -0.725   0.021   0.756   0.223
B A  2B42A                109    0.126  13.841  -2.612   0.023   0.655   0.322
B C  2B42A                110    0.098  13.759  -0.783   0.022   0.552   0.426
B A  2B42A                111    0.054   5.940  -1.239   0.021   0.451   0.528
E P  2B42A                112    0.298  42.286  -0.811   0.022   0.359   0.619
E S  2B42A                113    0.333  38.992  -1.202   0.066   0.296   0.638
B K  2B42A                114    0.278  57.102  -1.979   0.056   0.142   0.802
E L  2B42A                115    0.365  66.813  -2.674   0.113   0.043   0.844
B L  2B42A                116    0.316  57.805  -2.507   0.113   0.043   0.844
E A  2B42A                117    0.390  43.033  -1.922   0.184   0.043   0.773
E S  2B42A                118    0.367  43.059  -1.488   0.191   0.086   0.723
B L  2B42A                119    0.208  38.012  -1.526   0.191   0.086   0.723
E P  2B42A                120    0.396  56.136  -2.077   0.113   0.087   0.800
E R  2B42A                121    0.330  75.639  -1.611   0.113   0.087   0.800
E G  2B42A                122    0.348  27.356  -1.639   0.056   0.142   0.802
B S  2B42A                123    0.242  28.409  -2.614   0.056   0.142   0.802
E T  2B42A                124    0.321  44.509  -2.139   0.056   0.142   0.802
B G  2B42A                125    0.157  12.395  -2.203   0.064   0.216   0.721
B V  2B42A                126    0.218  33.568  -2.340   0.066   0.296   0.638
B A  2B42A                127    0.203  22.371  -2.175   0.066   0.296   0.638
B G  2B42A                128    0.228  17.944  -2.288   0.066   0.296   0.638
B L  2B42A                129    0.242  44.329  -2.014   0.066   0.296   0.638
E A  2B42A                130    0.318  35.088  -1.655   0.021   0.279   0.699
B N  2B42A                131    0.271  39.616  -1.001   0.020   0.205   0.775
E S  2B42A                132    0.374  43.880  -1.314   0.018   0.088   0.893
E G  2B42A                133    0.404  31.818  -1.721   0.052   0.084   0.864
B L  2B42A                134    0.209  38.359  -1.476   0.113   0.043   0.844
B A  2B42A                135    0.209  23.043  -2.637   0.181   0.016   0.803
E L  2B42A                136    0.316  57.786  -0.673   0.622   0.015   0.363
E P  2B42A                137    0.321  45.522  -1.948   0.717   0.014   0.269
E A  2B42A                138    0.289  31.837  -1.488   0.622   0.015   0.363
B Q  2B42A                139    0.187  33.434  -1.346   0.622   0.015   0.363
B V  2B42A                140    0.118  18.137  -1.151   0.622   0.015   0.363
E A  2B42A                141    0.387  42.680  -0.244   0.622   0.015   0.363
E S  2B42A                142    0.505  59.198  -0.912   0.522   0.016   0.462
E A  2B42A                143    0.484  53.381  -1.016   0.354   0.048   0.598
E Q  2B42A                144    0.441  78.763  -1.544   0.184   0.043   0.773
E K  2B42A                145    0.304  62.471  -0.863   0.113   0.087   0.800
B V  2B42A                146    0.272  41.760  -0.999   0.056   0.142   0.802
E A  2B42A                147    0.484  53.337  -0.334   0.019   0.141   0.840
E N  2B42A                148    0.293  42.837  -0.774   0.019   0.141   0.840
B R  2B42A                149    0.061  14.015   0.134   0.004   0.514   0.481
B F  2B42A                150    0.020   3.974   0.854   0.003   0.718   0.279
B L  2B42A                151    0.021   3.882   0.053   0.018   0.846   0.136
B L  2B42A                152    0.028   5.054   0.314   0.011   0.918   0.071
B C  2B42A                153    0.044   6.192  -0.341   0.018   0.846   0.136
B L  2B42A                154    0.033   5.969  -0.139   0.018   0.846   0.136
B P  2B42A                155    0.145  20.632  -0.291   0.004   0.616   0.381
B T  2B42A                156    0.233  32.275  -0.988   0.004   0.420   0.576
E G  2B42A                157    0.415  32.645  -1.243   0.019   0.141   0.840
E G  2B42A                158    0.504  39.649  -2.029   0.018   0.088   0.893
E P  2B42A                159    0.387  54.944  -1.579   0.019   0.141   0.840
B G  2B42A                160    0.258  20.336  -2.936   0.019   0.141   0.840
B V  2B42A                161    0.244  37.441  -1.077   0.021   0.279   0.699
B A  2B42A                162    0.113  12.420  -1.899   0.022   0.359   0.619
B I  2B42A                163    0.218  40.423  -0.970   0.022   0.359   0.619
B F  2B42A                164    0.102  20.491  -2.277   0.022   0.359   0.619
B G  2B42A                165    0.069   5.391  -3.947   0.022   0.359   0.619
B G  2B42A                166    0.176  13.883  -3.453   0.022   0.359   0.619
B G  2B42A                167    0.201  15.811  -1.555   0.022   0.359   0.619
B P  2B42A                168    0.275  38.994  -1.569   0.056   0.142   0.802
B V  2B42A                169    0.238  36.642  -0.773   0.052   0.084   0.864
E P  2B42A                170    0.321  45.522  -1.472   0.053   0.043   0.903
E W  2B42A                171    0.393  94.565  -0.971   0.058   0.017   0.925
E P  2B42A                172    0.515  73.107  -1.602   0.058   0.017   0.925
E Q  2B42A                173    0.467  83.460  -0.753   0.053   0.043   0.903
B F  2B42A                174    0.250  50.095  -1.394   0.018   0.088   0.893
E T  2B42A                175    0.514  71.278  -1.717   0.018   0.088   0.893
E Q  2B42A                176    0.421  75.119  -2.166   0.004   0.138   0.858
E S  2B42A                177    0.491  57.569  -0.738   0.004   0.197   0.799
B M  2B42A                178    0.117  23.372  -0.378   0.004   0.514   0.481
E P  2B42A                179    0.283  40.144  -0.548   0.002   0.816   0.182
B Y  2B42A                180    0.249  53.297  -0.517   0.002   0.816   0.182
B T  2B42A                181    0.076  10.500   0.542   0.002   0.816   0.182
B P  2B42A                182    0.169  23.939  -0.564   0.002   0.816   0.182
B L  2B42A                183    0.045   8.313  -0.556   0.003   0.718   0.279
B V  2B42A                184    0.132  20.304  -0.299   0.002   0.816   0.182
E T  2B42A                185    0.288  39.890  -0.388   0.004   0.514   0.481
E K  2B42A                186    0.518 106.614  -0.803   0.004   0.085   0.910
E G  2B42A                187    0.606  47.661  -2.701   0.005   0.015   0.979
E G  2B42A                188    0.623  49.014  -2.021   0.005   0.045   0.951
E S  2B42A                189    0.392  45.919  -1.126   0.004   0.138   0.858
E P  2B42A                190    0.314  44.599  -0.812   0.005   0.336   0.660
B A  2B42A                191    0.196  21.566  -1.196   0.003   0.718   0.279
B H  2B42A                192    0.091  16.589  -1.191   0.001   0.900   0.099
B Y  2B42A                193    0.221  47.142  -0.228   0.001   0.959   0.040
B I  2B42A                194    0.046   8.547  -0.272   0.001   0.959   0.040
B S  2B42A                195    0.226  26.464   0.270   0.001   0.959   0.040
B A  2B42A                196    0.055   6.072  -0.474   0.001   0.900   0.099
E R  2B42A                197    0.273  62.425  -0.335   0.001   0.900   0.099
B S  2B42A                198    0.175  20.487  -0.817   0.001   0.900   0.099
B I  2B42A                199    0.040   7.400  -1.196   0.001   0.900   0.099
B V  2B42A                200    0.174  26.775  -1.445   0.001   0.900   0.099
B V  2B42A                201    0.168  25.745  -1.086   0.003   0.718   0.279
E G  2B42A                202    0.425  33.487  -1.733   0.004   0.085   0.910
E D  2B42A                203    0.477  68.707  -2.105   0.005   0.045   0.951
E T  2B42A                204    0.538  74.662  -1.048   0.004   0.420   0.576
E R  2B42A                205    0.459 104.997  -1.419   0.004   0.514   0.481
B V  2B42A                206    0.178  27.328  -0.596   0.022   0.359   0.619
E P  2B42A                207    0.475  67.388  -1.576   0.021   0.279   0.699
B V  2B42A                208    0.207  31.801  -0.673   0.021   0.279   0.699
E P  2B42A                209    0.439  62.337  -1.285   0.020   0.205   0.775
E E  2B42A                210    0.388  67.801  -1.532   0.019   0.141   0.840
E G  2B42A                211    0.420  33.046  -1.792   0.019   0.141   0.840
B A  2B42A                212    0.299  32.895  -2.502   0.056   0.142   0.802
E L  2B42A                213    0.382  69.963  -2.097   0.052   0.084   0.864
E A  2B42A                214    0.459  50.538  -2.024   0.018   0.047   0.935
E T  2B42A                215    0.469  65.036  -1.058   0.018   0.047   0.935
E G  2B42A                216    0.334  26.270  -1.288   0.005   0.045   0.951
E G  2B42A                217    0.237  18.636   0.017   0.004   0.197   0.799
B V  2B42A                218    0.020   3.028   0.777   0.004   0.616   0.381
B M  2B42A                219    0.038   7.564  -0.190   0.001   0.900   0.099
B L  2B42A                220    0.019   3.516   0.587   0.001   0.900   0.099
B S  2B42A                221    0.050   5.848  -1.393   0.002   0.816   0.182
B T  2B42A                222    0.033   4.522  -1.616   0.020   0.205   0.775
B R  2B42A                223    0.225  51.571  -1.712   0.018   0.019   0.964
B L  2B42A                224    0.170  31.072  -0.518   0.018   0.047   0.935
B P  2B42A                225    0.138  19.525  -1.225   0.064   0.216   0.721
B Y  2B42A                226    0.155  33.145  -0.917   0.022   0.552   0.426
B V  2B42A                227    0.062   9.576  -1.852   0.022   0.552   0.426
B L  2B42A                228    0.155  28.381  -0.549   0.005   0.336   0.660
B L  2B42A                229    0.099  18.109  -1.138   0.020   0.205   0.775
B R  2B42A                230    0.149  34.006  -0.145   0.052   0.084   0.864
E P  2B42A                231    0.345  48.913  -0.444   0.802   0.014   0.185
E D  2B42A                232    0.539  77.627  -0.407   0.923   0.002   0.076
B V  2B42A                233    0.096  14.694  -0.796   0.970   0.001   0.030
B Y  2B42A                234    0.038   8.121  -0.615   0.970   0.001   0.030
E R  2B42A                235    0.417  95.447   1.137   0.970   0.001   0.030
E P  2B42A                236    0.276  39.136   1.171   0.970   0.001   0.030
B L  2B42A                237    0.027   5.035   0.338   0.970   0.001   0.030
B M  2B42A                238    0.138  27.594   0.702   0.970   0.001   0.030
E D  2B42A                239    0.563  81.071   0.903   0.988   0.000   0.012
B A  2B42A                240    0.217  23.957   0.076   0.970   0.001   0.030
B F  2B42A                241    0.038   7.707  -0.551   0.970   0.001   0.030
E T  2B42A                242    0.384  53.261   0.307   0.923   0.002   0.076
E K  2B42A                243    0.505 103.817  -0.535   0.923   0.002   0.076
B A  2B42A                244    0.249  27.418  -0.590   0.879   0.010   0.111
B L  2B42A                245    0.169  30.962  -1.325   0.802   0.014   0.185
E A  2B42A                246    0.563  61.999  -0.889   0.622   0.015   0.363
E A  2B42A                247    0.479  52.731  -1.310   0.339   0.016   0.645
E Q  2B42A                248    0.469  83.817  -1.249   0.184   0.043   0.773
E G  2B42A                249    0.475  37.359  -1.699   0.113   0.043   0.844
E A  2B42A                250    0.357  39.297  -1.079   0.056   0.142   0.802
E P  2B42A                251    0.423  60.052  -1.759   0.064   0.216   0.721
E V  2B42A                252    0.313  48.139  -1.126   0.020   0.205   0.775
E A  2B42A                253    0.359  39.540  -1.254   0.020   0.205   0.775
E R  2B42A                254    0.451 103.187  -1.375   0.020   0.205   0.775
E A  2B42A                255    0.417  45.909  -1.225   0.056   0.142   0.802
E V  2B42A                256    0.298  45.833  -1.821   0.052   0.084   0.864
E V  2B42A                257    0.441  67.828  -1.814   0.113   0.043   0.844
E A  2B42A                258    0.510  56.213  -1.803   0.113   0.043   0.844
E V  2B42A                259    0.394  60.496  -1.823   0.115   0.016   0.868
E A  2B42A                260    0.488  53.800  -1.529   0.113   0.043   0.844
E P  2B42A                261    0.394  55.866  -1.593   0.053   0.043   0.903
B F  2B42A                262    0.232  46.603  -1.604   0.052   0.084   0.864
E G  2B42A                263    0.394  31.016  -0.920   0.056   0.142   0.802
B V  2B42A                264    0.231  35.459  -0.574   0.020   0.205   0.775
B C  2B42A                265    0.069   9.758  -2.547   0.021   0.279   0.699
B Y  2B42A                266    0.106  22.567  -1.035   0.022   0.359   0.619
E D  2B42A                267    0.407  58.692  -1.376   0.022   0.359   0.619
B T  2B42A                268    0.204  28.309  -1.134   0.020   0.205   0.775
E K  2B42A                269    0.475  97.646  -1.671   0.020   0.205   0.775
E T  2B42A                270    0.401  55.660  -1.183   0.021   0.279   0.699
E L  2B42A                271    0.380  69.505  -1.717   0.020   0.205   0.775
B G  2B42A                272    0.279  21.942  -1.628   0.019   0.141   0.840
E N  2B42A                273    0.475  69.467  -1.231   0.019   0.141   0.840
B N  2B42A                274    0.274  40.143  -1.611   0.018   0.088   0.893
E L  2B42A                275    0.518  94.846  -1.386   0.018   0.088   0.893
E G  2B42A                276    0.536  42.175  -1.314   0.018   0.088   0.893
E G  2B42A                277    0.325  25.554  -0.640   0.018   0.088   0.893
E Y  2B42A                278    0.437  93.408  -1.705   0.020   0.205   0.775
E A  2B42A                279    0.441  48.554  -0.654   0.020   0.205   0.775
B V  2B42A                280    0.118  18.121  -0.045   0.004   0.138   0.858
B P  2B42A                281    0.151  21.370  -0.307   0.004   0.085   0.910
E N  2B42A                282    0.311  45.486  -0.350   0.004   0.420   0.576
B V  2B42A                283    0.053   8.208   0.269   0.002   0.816   0.182
B Q  2B42A                284    0.196  35.077  -0.130   0.001   0.900   0.099
B L  2B42A                285    0.039   7.068  -0.858   0.001   0.959   0.040
E G  2B42A                286    0.261  20.541   0.409   0.001   0.959   0.040
B L  2B42A                287    0.077  14.135  -0.530   0.001   0.900   0.099
E D  2B42A                288    0.409  58.937   0.416   0.005   0.262   0.733
E G  2B42A                289    0.595  46.850  -2.369   0.005   0.015   0.979
E G  2B42A                290    0.511  40.192  -1.750   0.004   0.138   0.858
E S  2B42A                291    0.271  31.714  -0.037   0.003   0.718   0.279
E D  2B42A                292    0.494  71.157   0.242   0.001   0.900   0.099
B W  2B42A                293    0.100  24.170   1.161   0.001   0.900   0.099
E T  2B42A                294    0.452  62.679  -0.139   0.001   0.900   0.099
B M  2B42A                295    0.069  13.807  -0.324   0.003   0.718   0.279
E T  2B42A                296    0.359  49.752   0.825   0.004   0.197   0.799
B G  2B42A                297    0.258  20.336  -1.432   0.018   0.047   0.935
E K  2B42A                298    0.525 107.992  -0.577   0.019   0.141   0.840
B N  2B42A                299    0.235  34.419  -1.851   0.021   0.279   0.699
B S  2B42A                300    0.064   7.513   0.117   0.022   0.552   0.426
B M  2B42A                301    0.069  13.767  -1.121   0.018   0.846   0.136
B V  2B42A                302    0.117  18.014  -0.150   0.011   0.918   0.071
E D  2B42A                303    0.266  38.316  -0.426   0.011   0.918   0.071
B V  2B42A                304    0.216  33.168  -0.396   0.023   0.655   0.322
E K  2B42A                305    0.486 100.052  -1.964   0.005   0.336   0.660
E Q  2B42A                306    0.684 122.162  -2.373   0.005   0.045   0.951
E G  2B42A                307    0.644  50.706  -2.622   0.005   0.015   0.979
E T  2B42A                308    0.370  51.388  -0.813   0.004   0.085   0.910
B A  2B42A                309    0.126  13.918  -0.044   0.004   0.514   0.481
B C  2B42A                310    0.042   5.841  -0.441   0.004   0.616   0.381
B V  2B42A                311    0.027   4.135   0.524   0.002   0.816   0.182
B A  2B42A                312    0.013   1.455   0.466   0.001   0.900   0.099
B F  2B42A                313    0.020   3.934   0.424   0.001   0.900   0.099
B V  2B42A                314    0.022   3.366  -0.237   0.018   0.846   0.136
B E  2B42A                315    0.053   9.189  -2.075   0.022   0.552   0.426
B M  2B42A                316    0.054  10.725  -2.073   0.021   0.279   0.699
B K  2B42A                317    0.199  40.893  -0.731   0.052   0.084   0.864
B G  2B42A                318    0.258  20.320  -2.334   0.053   0.043   0.903
B V  2B42A                319    0.264  40.592  -2.134   0.053   0.043   0.903
E A  2B42A                320    0.414  45.645  -2.629   0.053   0.043   0.903
B A  2B42A                321    0.287  31.649  -2.263   0.018   0.047   0.935
E G  2B42A                322    0.521  40.987  -2.520   0.018   0.047   0.935
E D  2B42A                323    0.438  63.130  -1.928   0.018   0.019   0.964
E G  2B42A                324    0.590  46.433  -3.016   0.018   0.019   0.964
E R  2B42A                325    0.531 121.691  -2.544   0.018   0.019   0.964
E A  2B42A                326    0.352  38.845  -1.019   0.018   0.019   0.964
E P  2B42A                327    0.286  40.569  -0.487   0.053   0.043   0.903
B A  2B42A                328    0.085   9.400  -0.229   0.069   0.386   0.545
B V  2B42A                329    0.061   9.345  -0.115   0.085   0.775   0.140
B I  2B42A                330    0.024   4.514   0.459   0.018   0.846   0.136
B L  2B42A                331    0.034   6.152   0.053   0.172   0.690   0.138
B G  2B42A                332    0.036   2.865  -0.806   0.538   0.173   0.289
B G  2B42A                333    0.116   9.145  -0.311   0.802   0.014   0.185
B A  2B42A                334    0.178  19.572   0.063   0.751   0.050   0.199
B Q  2B42A                335    0.076  13.609  -0.380   0.660   0.049   0.291
B M  2B42A                336    0.054  10.765  -0.205   0.660   0.049   0.291
E E  2B42A                337    0.261  45.509   0.598   0.354   0.048   0.598
E D  2B42A                338    0.272  39.224  -0.642   0.118   0.150   0.732
B F  2B42A                339    0.061  12.203   0.662   0.023   0.655   0.322
B V  2B42A                340    0.061   9.437   0.787   0.011   0.918   0.071
B L  2B42A                341    0.030   5.420   0.706   0.006   0.962   0.032
B D  2B42A                342    0.030   4.395   0.250   0.006   0.962   0.032
B F  2B42A                343    0.057  11.440   1.236   0.006   0.962   0.032
B D  2B42A                344    0.096  13.776   0.164   0.018   0.846   0.136
E M  2B42A                345    0.241  48.304   0.488   0.005   0.262   0.733
E E  2B42A                346    0.625 109.135   0.240   0.005   0.045   0.951
E K  2B42A                347    0.607 124.860   0.115   0.005   0.015   0.979
E K  2B42A                348    0.407  83.658  -0.068   0.018   0.047   0.935
B R  2B42A                349    0.205  47.037   0.897   0.003   0.718   0.279
B L  2B42A                350    0.028   5.072   0.484   0.011   0.918   0.071
B G  2B42A                351    0.061   4.801  -0.528   0.011   0.918   0.071
B F  2B42A                352    0.042   8.409  -0.418   0.011   0.918   0.071
B S  2B42A                353    0.044   5.157  -0.349   0.018   0.846   0.136
E R  2B42A                354    0.352  80.654  -0.209   0.023   0.655   0.322
B L  2B42A                355    0.155  28.417   0.036   0.021   0.279   0.699
E P  2B42A                356    0.525  74.455  -0.478   0.020   0.205   0.775
E H  2B42A                357    0.348  63.265   0.250   0.019   0.141   0.840
E F  2B42A                358    0.507 101.755  -1.739   0.018   0.088   0.893
E T  2B42A                359    0.306  42.456  -1.361   0.018   0.047   0.935
E G  2B42A                360    0.556  43.765  -0.665   0.018   0.047   0.935
E C  2B42A                361    0.312  43.763  -1.943   0.018   0.047   0.935
E G  2B42A                362    0.389  30.622  -1.773   0.018   0.019   0.964
E G  2B42A                363    0.474  37.335  -1.412   0.018   0.019   0.964
E L  2B42A                364    0.392  71.702  -1.441   0.003   0.003   0.994

Explain the output.  Go back.
```

---
